# Simultaneous Screening of Major Flame Retardants and Plasticizers in Polymer Materials Using Pyrolyzer/Thermal Desorption Gas Chromatography Mass Spectrometry (Py/TD–GC–MS)

**DOI:** 10.3390/molecules23040728

**Published:** 2018-03-22

**Authors:** Hiroyuki Yanagisawa, Yukihiko Kudo, Katsuhiro Nakagawa, Haruhiko Miyagawa, Fumitaka Maruyama, Shigehiko Fujimaki

**Affiliations:** 1 Consumer & Retail Service Division, SGS Japan Inc. YBP East Tower 12F, 134 Godo-cho, Hodogaya-ku, Yokohama 240-0005, Japan; hiroyuki.yanagisawa@sgs.com (H.Y.); fumitaka.maruyama@sgs.com (F.M.); 2 Analytical & Measuring Instrument Division, Shimadzu Corporation, 1 Nishinokyo Kuwabara-cho, Nakagyo-ku, Kyoto 640-8511, Japan; yuk-kudo@shimadzu.co.jp (Y.K.); nk@shimadzu.co.jp (K.N.); miyagawa@shimadzu.co.jp (H.M.)

**Keywords:** additives, short chain chlorinated paraffins, decabromodiphenyl ether, hexabromocyclododecane, di(2-ethylhexyl) phthalate, pyrolysis, thermal desorption, plasticizer, HBCDD, DEHP

## Abstract

This study was conducted with the aim of achieving the simultaneous screening of various additives in polymer materials by utilizing a solvent-free pyrolyzer/thermal desorption gas chromatography mass spectrometry (Py/TD-GC–MS) method. As a first step to achieve this goal, simultaneous screening has been examined by selecting major substances representing plasticizers and flame retardants, such as short chain chlorinated paraffins (SCCPs), decabromodiphenyl ether (DecaBDE), hexabromocyclododecane (HBCDD), and di(2-ethylhexyl) phthalate (DEHP). A quantitative MS analysis was performed to check for the peak areas and sensitivities. Since Py/TD-GC–MS is fraught with the risk of thermal degradation of the sample, temperatures during the analytical process were finely tuned for securing reliable results. The instrumental sensitivity was confirmed by the S/N ratio on each component. The detection limits of all components were less than 50 mg/kg, which are sufficiently lower than the regulatory criteria. With regard to reproducibility, a relative standard deviation (RSD) of about 5% was confirmed by employing a spike recovery test on a polystyrene polymer solution containing mixed standard solution (ca. 1000 mg/kg). In conclusion, the results obtained in this study indicate that Py/TD-GC–MS is applicable for the screening of major flame retardants and plasticizers in real samples with sufficient reproducibility at regulatory levels.

## 1. Introduction

Plastics are chemical products manufactured from various petroleum-based feedstocks. Many convenient products have been made from plastics over the past few decades. Advances in additive technology have made plastic products extremely useful and widespread.

In contrast, the use of certain additives has recently become a controversial issue since some additives have been found to be toxic to human beings [1,2,3,4,5]. For instance, certain types of plasticizers and flame retardants are restricted by international laws on chemicals [6,7,8]. Moreover, the ban on the use of harmful substances is becoming more strictly enforced under such laws [9]. In this regard, special attention must be paid to certain types of plasticizers and flame retardants. Plasticizers and flame retardants are often compounded at a high concentration, usually in the 1 to 30% range [5,10], and can pose a threat to human health as reproductive toxicants, persistent organic pollutants, and carcinogens [11,12,13].

One problem is that the inclusion of such additives cannot be detected without detailed analyses. Test methods such as GC–MS analysis can be used to identify and quantify such additives [14,15], however, the conventional GC–MS method requires prolonged pre-treatment procedures. For example, the solvent extraction process is sometimes complicated or difficult to perform. In other words, the presence or absence of hazardous substances can be determined only by expert analysis, which is usually expensive and time consuming [14,15].

Under such circumstances, the concept of screening has recently been discussed with a view to avoiding the complexity of GC–MS analysis [16]. Specifically, pyrolysis gas chromatography mass spectrometry (Py-GC–MS) was proposed as a way of screening certain phthalates as an alternative to conventional GC–MS. The proposed screening method employs direct sample injection and thermal extraction in the GC–MS analysis and does not require any complicated pre-treatment using organic solvents [17,18,19,20]. Py-GC–MS was originally used for identifying polymers from the pattern of peaks obtained in a chromatogram [21,22,23]. Recently, another application of Py-GC–MS has been attracting attention from a different perspective [18,19,20,24,25]. Py-GC–MS is reportedly beneficial when screening for additives in polymer materials [26]. The analysis of thermally desorbed compounds by Py-GC–MS is often referred to as the pyrolyzer/thermal desorption (Py/TD-GC–MS) method.

In pursuit of a convenient screening approach, the International Electrotechnical Commission (IEC) has recently published a method using Py/TD-GC–MS as part of a new IEC-62321-8 standard [16,19,20,27]. Although many studies have reported methods for analyzing phthalates, IEC62321-8 is so far the only international standard that defines the screening of certain restricted phthalates. The screening approach is used to determine the presence or absence of an analyte based on a comparison with a preset threshold. However, the current IEC screening method only focuses on certain phthalates and does not include any other additives. As mentioned above, along with certain plasticizers, other additives, such as brominated flame retardants, are also subject to international chemical regulation [28,29]. These additives are now labeled as hazardous substances and are regulated by international chemical use laws.

This research is intended to explore further possibilities of the test method that was originally standardized as IEC62321-8 for the screening of certain phthalates. In this study, Py/TD-GC–MS conditions presented in IEC62321-8 came under review from the perspective of simultaneous screening. The purpose of this study is to demonstrate the benefit of a screening method that utilizes the unique features of Py/TD-GC–MS to identify harmful additives such as plasticizers and flame retardants. As a first step to achieve this goal, simultaneous screening has been examined by selecting major substances representing flame retardants and plasticizers such as SCCPs, DecaBDE, HBCDD, and DEHP.

Among these substances, SCCPs are generally a mixture of multiple congeners and isomers and require special attention when identifying their inclusion in polymer materials [30]. In this respect, the screening of SCCPs is regarded as highly challenging and has become a major research focus [31].

For the screening of brominated flame retardants, suppression of thermal degradation is an essential requirement for obtaining reliable analytical results [32]. Therefore, maintaining finely tuned temperatures during the sample injection and GC separation is regarded as another challenge of this study.

With this in mind, we studied a simple way to determine SCCPs and brominated flame retardants as part of the larger aim of achieving the simultaneous screening of various additives in polymer materials using Py/TD-GC–MS. It should be noted that the scope of this study is limited to the screening of thermally extractable compounds detected with an electron ionization (EI)-MS unit after chromatographic separation with a GC column. In addition, simultaneous screening is performed with a constant condition that is not necessarily optimized for the quantification of each individual substance.

Despite such limitations, by applying a practical safety margin to regulatory criteria as a precaution to avoid misinterpretation of analytical results in determining the presence/absence of regulated additives, we found our proposed screening method using Py/TD-GC–MS to be effective in verifying compliance with major chemical regulations.

## 2. Results

Although precise quantification of each individual substance is not a major subject in this study, the method has to be adjusted to ensure a fast analysis at moderate resolution, sensitivity, and detection limits. Therefore, we carefully examined key experimental parameters and obtained the following results.

### 2.1. MS Quantification

**Figure 1:**
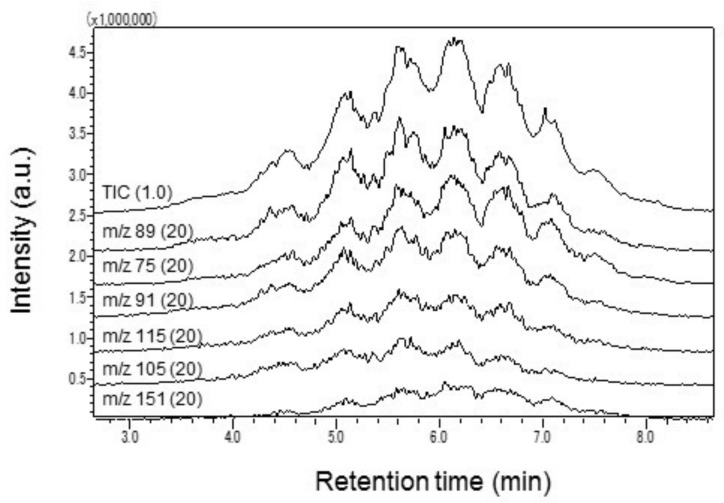
Major ion signals specific to fragments of a SCCP standard solution. Ion current intensities, except for total ion chromatogram (TIC), are enlarged by 20 times. Ion signals are displayed to the same scale of vertical axis by shifting the base line. Numbers in parentheses indicate the magnification.

All targeted analytes need to be identified based on the GC retention times and mass spectra. In the MS quantitative analysis, signals are analyzed by focusing on a specific ion, which is often called as a target ion. The selection of an appropriate target ion is particularly crucial in MS data analysis. In general, strong signal ions in a relatively high *m*/*z* region are used for quantitative mass analysis to avoid interference noises. However, it is sometimes quite difficult to come across an appropriate ion for the MS quantification, especially if multiple isomers are involved. For example, in case of SCCPs, EI produces mass spectra of only weak ion signals in the upper *m*/*z* region. Thus, previous studies have documented the effectiveness of GC-negative ion chemical ionization (NICI)-MS in SCCPs data analysis [33,34,35]. Since Py/TD-GC–MS is operated with EI-MS, ions with a relatively strong SCCP related signal only appeared in the lower *m*/*z* region. For this reason, these ions were selected for the identification and quantification of SCCPs by carefully examining the interference noise levels of typical polymer matrices. Figure 1 shows the characteristic profiles obtained over a wide bandwidth (about 3 to 8 min in retention time) in a mass chromatogram of 1000 mg/kg SCCPs.

**Figure 2:**
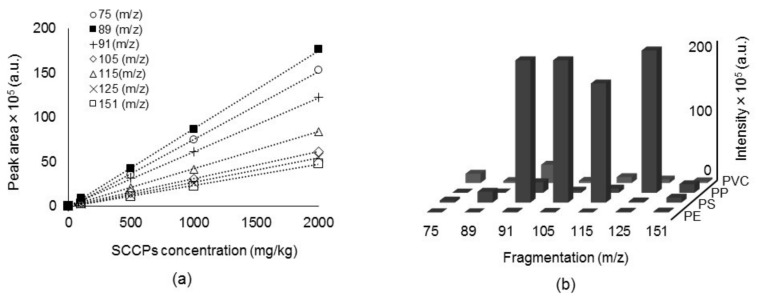
(**a**) Comparison of calibration curves of individual ion signals for SCCPs analysis. Area represents the average of three repeated measurements; (**b**) Overlapping noise levels of various polymer materials. PE: polyethylene, PS: polystyrene, PP: polypropylene, PVC: polyvinyl chloride.

Relatively strong ion signals at *m*/*z* 75, 89 were commonly found that were specific to fragments of SCCP standard solutions. Besides the two predominant ions, signals at *m*/*z* 91, 105, 115, 125, and 151 were also found in the EI fragmentation of the SCCPs. Herein, a quantitative selected ion monitoring (SIM) analysis was performed over a wide bandwidth to cover all SCCPs related peaks and to compare the sensitivities of individual ions. As can be seen in Figure 2a and Supplementary Material Table S1, the ion signal sensitivities were in the order *m*/*z* 89 > 75 > 91 > 105 > 115 > 151. Previous studies selected a signal at *m*/*z* 91 (C_4_H_8_Cl) or 105 (C_5_H_10_Cl) as the target ion for the quantification of SCCPs [36]. When selecting a target ion in the lower *m*/*z* region, overlapping noise levels on each ion signal ought to be examined in detail to secure a reliable result. In this regard, overlapping noise levels in the SCCP peak area were examined with various polymer materials and the results are summarized in Figure 2b. Of all the ion signals, those at *m*/*z* 75 and 89 were less influenced by the overlapping background signal. In view of this result, the ion signal at *m*/*z* 89, rather than at *m*/*z* 91 or 105, was selected as the target ion for SCCP quantification.

Except for SCCPs, there was no such complexity in finding a characteristic ion for the target analyte. Abundant ions characteristic to each component have been selected as target ions from the spectrum obtained by injecting standard samples. Mass Spectral Databases included in the GC–MS system (NIST11 and NIST11s) were used to facilitate compound identification. All the target ions used for the mass analyses are summarized in Table 1, together with specific ions for the confirmation of each component.

### 2.2. Method Optimization

#### 2.2.1. Py/TD Process

The sample was placed in a Py/TD furnace that could be heated rapidly to a sufficiently high temperature to produce a thermal desorption of a wide variety of compounds. To achieve meaningful results, the heating temperatures must be controlled below 350 °C at which the excessive decomposition of base polymers can be suppressed.

**Figure 3:**
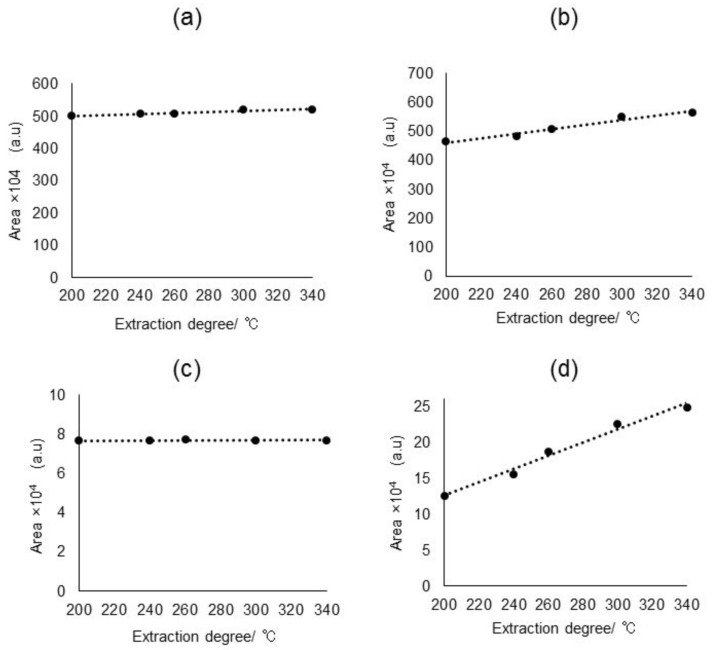
Temperature dependent extraction efficiencies of 1000 mg/kg (**a**) SCCPs; (**b**) DEHP; (**c**) HBCDD, and (**d**) DecaBDE in Py/TD process under a constant GC–MS condition.

In this study, the Py/TD settings have been narrowed down for the simultaneous screening by referring to the conditions specified in IEC62321-8 [16]. Heating temperature of up to 340 °C has been examined in detail with respect to all the analytes that are involved in this study. As can been seen in Figure 3, a relatively higher temperature up to 340 °C was proven to be suitable to ensure a sufficient MS peak area of DecaBDE and did not create any adverse impact on other analytes. Finally, volatile compounds released from the sample under the specified heating condition were transferred to a GC–MS for screening analysis.

#### 2.2.2. GC Conditions

As for the GC process, a 15 m Ultra ALLOY-PBDE (UA-PBDE) column was used to rapidly separate multiple components by differences in boiling points. The UA-PBDE column is a very inert metal capillary tube with highly deactivated inner wall and ultra-thin stationary phase film specifically designed for minimizing absorption and thermal decomposition of brominated flame retardants.

After the GC column selection, column oven temperature is considered as one of the most important variables in GC separation. In general, column temperature should be high enough so that sample components pass through the column at reasonable speed, but should not be too high to avoid thermal degradation of the sample and facilitate better resolution. In addition, the process of increasing the column temperature during a GC separation is commonly employed as a very effective method for optimizing an analysis of complex samples. If a sample being analyzed contains components whose vapor pressure (boiling points) extend over a wide range, temperature programming should be used to obtain optimum separation in the minimum time. The initial and final column temperatures need to be set to allow elution of all analytes at a constant temperature program rate within a preset time, including the hold time at the end of the run for the elution of extraneous compounds.

**Figure 4:**
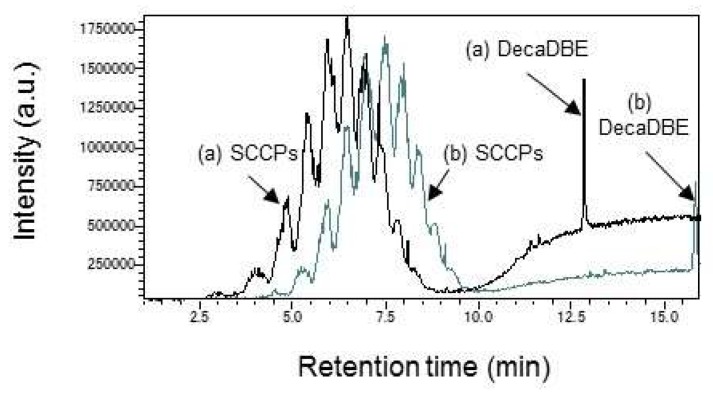
TIC Chromatogram using Py/TD–GC–MS at SCCPs and DecaBDE. Black line (a): the initial and final column temperatures are 80 to 300 °C at 20 °C/min. Gray line (b): 60 to 280 °C at 20 °C/min. GC was run by the preconditioned sample conditions except for the column temperature.

In this study, a high temperature program rate of 20 °C/min was employed to minimize the time of analytical run while securing the retention time reproductivity. The column temperature was increased from 80 to 300 °C at 20 °C/min and held at 300 °C for 5 min during a GC separation. As shown in Figure 4, lower column temperatures (e.g., the initial and final column temperatures of 60 and 280 °C, respectively) delayed the elution of DecaBDE by about 2.5 min and were not good enough for all analytes to pass through the column within the preset time.

### 2.3. Calibration Curves and Detection Limits

**Figure 5:**
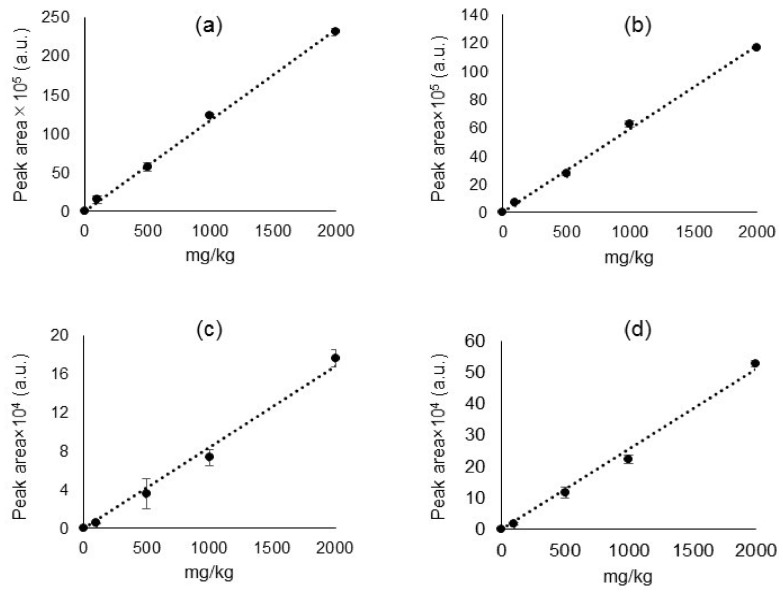
Calibration curves of (**a**) DEHP (*m*/*z* 279); (**b**) SCCPs (*m*/*z* 89); (**c**) HBCDD (*m*/*z* 319), and (**d**) DecaBDE (*m*/*z* 799), each 5-point calibration by 0, 100, 500, 1000, and 2000 mg/kg. Error bars represent the average of three repeated measurements.

A quantitative SIM analysis was performed on all target ions to check for the peak areas and sensitivities of individual ions. The results are plotted in Figure 5 as the calibration curves of individual ion signals. Sensitivities (i.e., slope of calibration curve), correlation coefficients (R^2^), relative standard deviations (RSD), and detection limits are summarized in Table 2. The calibration curves were all linear with excellent R^2^ values of over 0.98 and about 5% RSD. At the same time, detection limit (LOD) study was conducted with the selected ion signals listed in Table 1. The instrumental sensitivity was confirmed by the S/N ratio of 50 ng (equivalent to 100 mg/kg) on each component. The LODs of SCCPs, DecaBDE, HBCDD, and DEHP were less than 50 mg/kg, as determined by repeating the measurement 7 times, as shown in Supplementary Material Table S2 (Student’s *t*-test with 99% confidence), which is sufficiently lower than the regulatory criteria for these restricted substances of 1000–1500 mg/kg [6,7,8,9].

### 2.4. Analysis of Complex Samples

**Figure 6:**
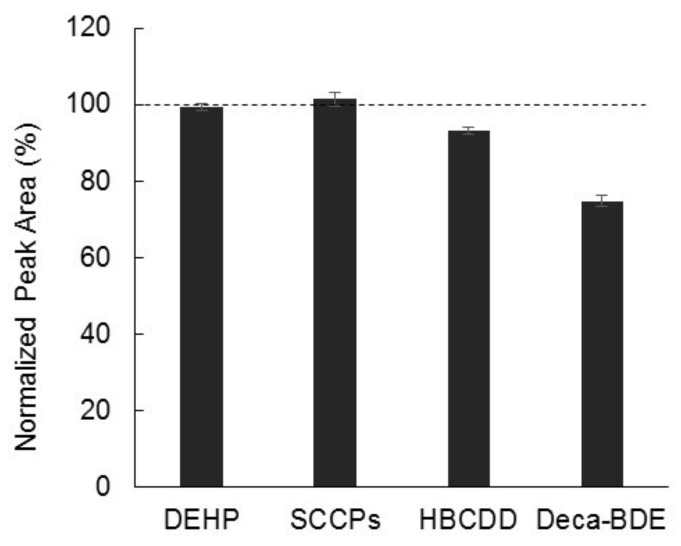
Results of the spike test with the addition of 1000 mg/kg mixed standard to the polystyrene solution. The value of each analyte is expressed relative to the concentration of the mixed standard. Error bars represent one standard deviation of three repeated measurements.

In the next stage, we conducted an empirical assessment with regards to application to real samples by referring to the abovementioned preliminary studies. Since certified reference materials are not commercially available for all the analytes, a real sample was needed to demonstrate the applicability of Py/TD-GC–MS to the simultaneous screening (by Py/TD-GC–MS). However, it is not easy to find a positive sample containing all the analytes. Instead, simulant samples were prepared as alternatives to real samples. A simulant sample was prepared by adding a mixed solution to a polystyrene polymer solution and then drying the sample at room temperature. Analyte concentrations of mixed standard solution (ea. 1000 mg/kg) were quantified by the Py/TD-GC–MS simultaneous screening (*n* = 3). As can be seen in Figure 6, results of the spike test were close to the setup value within an acceptable margin (>75%), and the reproducibility was about RSD 5% for all the analytes. These results indicate that major additives present in polymer materials can be simultaneously determined by Py-TD-GC–MS.

## 3. Discussion

This study was conducted with the aim of achieving the simultaneous screening of various additives in polymer materials using a Py/TD-GC–MS system. In this regard, the scope of this study is limited to the screening of thermally extractable compounds that are detected with EI-MS after chromatographic separation with a GC column.

It should be noted that simultaneous screening is performed with a constant condition that is not necessarily optimized to the quantification of each individual substances. In other words, screening by the Py/TD-GC–MS method is subject to certain limitations in ionization mode, GC column, and temperature controls.

**Figure 7:**
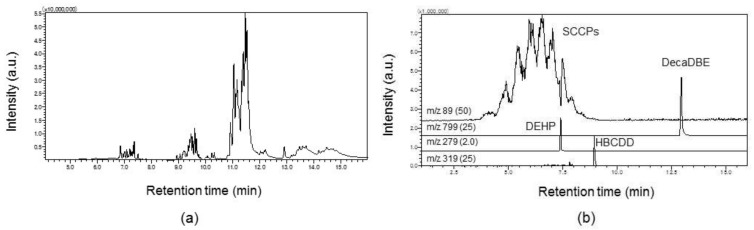
Simultaneous screening of four additives contained in polystyrene at a concentration of 1000 mg/kg. (**a**) TIC; (**b**) SIM for DEHP (*m*/*z* 279), SCCPs (*m*/*z* 89), HBCDD (*m*/*z* 319), and DecaBDE (*m*/*z* 799). Target ion signals are displayed to the same scale of the vertical axis by shifting the base line. Numbers in parentheses indicate the magnification.

This was especially crucial for the determination of SCCPs. Relatively strong SCCP related ion signals appeared only in the “lower *m*/*z*” region wherein background noises are predominant. Therefore, target ions for MS analysis have been selected with great care. In this study, the ion signal at *m*/*z* 89 has been selected as a target ion to secure sufficient S/N ratio for the quantification of SCCPs in most major polymer matrices. As described above, finding an ion specific to each individual additive is the key to achieving simultaneous screening. This is not limited to the screening of SCCPs. It is also true in the screening of other additives. Figure 7 shows an example of simultaneous screening of typical flame retardants and plasticizers in polystyrene. In case of GC–MS, separation may not be so important if the coeluting compounds have different mass spectra. As evident from Figure 7b, all target ions have been measured without being interfered with each other by selecting appropriate quantitative ions. In addition, LOD of each component has been confirmed to be about 50 mg/kg and the results are summarized in the Supplementary Material (Table S3).

These ion signals are individually quantified by SIM data analysis. However, data in Figure 6 suggest that brominated flame retardants are somewhat subjected to thermal degradation under a certain circumstance. Thermal degradation is likely to occur in brominated flame retardants via debromination [23,24]. It is of particular concern in the DecaBDE but also occurs in the HBCDD. Degradation of HBCDD is considered to become significant at temperatures above 240 °C [37]. Therefore, an appropriate margin for screening with reference to the regulatory criteria has been proposed as a sensible precaution to avoid being deceived by the misinterpretation of thermally degraded peaks. For example, a preset margin of 50% to the regulatory limit should help to facilitate the determination of presence or absence of restricted additives by the screening method. In this case, values below 500 mg/kg and over 1500 mg/kg can be regarded as “below limit” and “over limit”, respectively, with regard to the regulatory limit of 1000 mg/kg. However, if unfortunately values between 500 and 1500 mg/kg were produced by the screening method, follow-up actions would be recommended to make a final presence/absence decision by applying conventional GC–MS or LC–MS methods.

Although there still remain some technical limitations, the proposed screening approach should help reduce the burden of verifying compliance with chemical regulations by taking preventative measures based on an understanding of the unique features of the Py/TD-GC–MS method.

## 4. Materials and Methods

The purpose of this study is to offer a quick and efficient way to screen harmful additives in polymer materials and help reduce the burden of confirming compliance with chemical regulations. In this study, we examine and demonstrate the application of Py/TD-GC–MS to the simultaneous screening of major flame retardants and phthalate. The initial setting for the analysis was selected with reference to the screening step of the IEC62321-8 standard [16].

### 4.1. Reagents and Materials

Chlorowax 500C (AccuStandard Inc., New Haven, CT, USA; 100 mg/kg in toluene with an average chain length of C_12_ and 59% chlorination) was used as an SCCP standard mixture to represent carcinogenic SCCP. The SCCP standard solution was selected in accordance with findings on carcinogenicity published by the International Agency for Research on Cancer (IARC, Lyon, France). IARC classifies SCCPs with an average chain length of C_12_ and 60% chlorination as being possibly carcinogenic to human beings [13,38]. Other standard reagents, such as DecaBDE, HBCDD and DEHP (Tokyo Chemical Industry Co., Ltd., Tokyo, Japan), were also used in this study.

The materials used in this study include the following: polypropylene powder (isotactic, average M_w_ 12,000, catalog number 428116, Sigma-Aldrich Co. LLC, St. Louis, MO, USA), polyvinyl chloride powder (low molecular weight, catalog number 81388, Sigma-Aldrich Co. LLC), and polystyrene powder (average M_w_ 35,000, catalog number 331651, Sigma-Aldrich Co. LLC). Other reagents, namely ethanol (99.5%) and toluene (for high performance liquid chromatography; all analytical grade, Kanto Chemical Co., Inc., Tokyo, Japan), were also obtained for this study. Simulant samples were prepared by adding a mixed solution to a polymer solution and then drying the sample at room temperature.

### 4.2. Preparation of Mixture Samples for Simultaneous Analysis

By assuming the sample weight of 0.5 mg in Py/TD-GC–MS, each standard solution was diluted with toluene to produce calibration curves in the 0 to 2000 mg/kg concentration range. As an example, 5 μl amounts of standard solutions containing SCCPs, DecaBDE, HBCDD, and DEHP at a concentration of 100 mg/kg were injected into the sample cup to demonstrate simultaneous screening with Py/TD-GC–MS. In this case, SCCPs (*m*/*z* 89), DecaBDE (*m*/*z* 799), HBCDD (*m*/*z* 319), and DEHP (*m*/*z* 270) were identified as 1000 mg/kg equivalent in a chromatogram.

### 4.3. Instrument Setup

**Figure 8:**
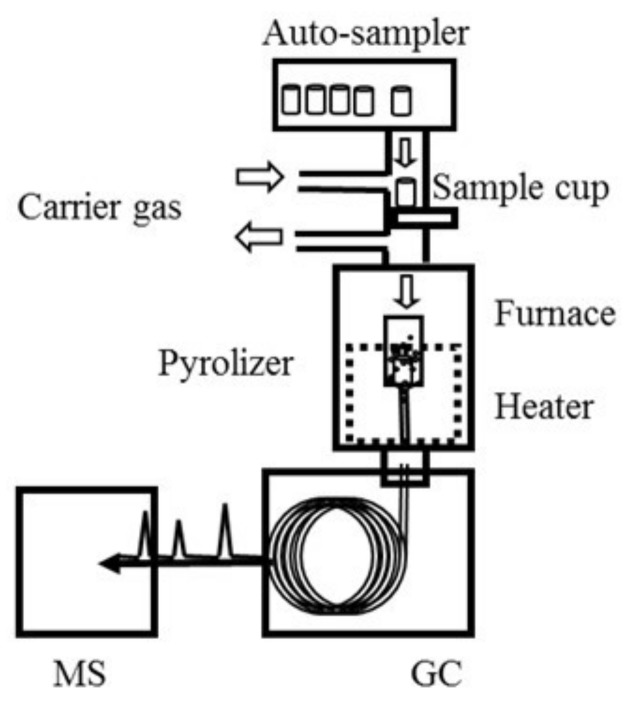
Schematic of the Py/TD-GC–MS system.

Major flame retardants and phthalate were analyzed using a GC–MS system (GC–MS-QP2010 Ultra, Shimadzu Corp., Kyoto, Japan) equipped with a pyrolyzer/thermal desorption (Py/TD) unit (EGA/PY-3030D, Frontier Lab, Koriyama, Japan) mounted on top of the GC inlet. Figure 8 is a schematic of the Py/TD-GC–MS system. Samples were placed in sample cups and then directly dropped into the furnace attached to the inlet of the GC unit for thermal desorption under a specific heat zone. Thermally desorbed gaseous components were then transferred to the gas chromatography unit, separated by a capillary column and detected with a mass spectrometer. In addition, the use of an auto-sampler (AS-1020E, Frontier Lab) enabled us to perform a sequential testing cycle that lasted about 30 min per sample.

### 4.4. Py/TD-GC–MS Parameter Settings

It should be noted that interference from matrix components could cause a serious problem when analyzing additives in polymers. According to the IEC 62321-8 test standard [16], phthalates are thermally extracted under a specific heating condition to avoid the major decomposition of base materials. Especially, the SCCPs were carefully analyzed to minimize interference from base materials. The typical Py/TD heating condition and GC–MS parameter settings are listed in Table 3. A 15 m × 0.25 mm I.D. GC column and a 0.05-µm thick film (Ultra ALLOY-PBDE, 100% dimethyl polysiloxane, Frontier Lab) were used for the measurement. The column temperature was increased from 80 to 300 °C at 20 °C/min and held at 300 °C for 5 min during a GC run. The injection mode was set to split with a ratio of 1/50, and helium (purity of greater than a volume fraction of 99.999%) was used as a carrier gas at a constant linear velocity of 52.1 cm/s. A mass analysis was performed using both SIM and TIM modes. A specific mass range from *m*/*z* 50 to 1000 is scanned by TIM. The ion source was controlled at 230 °C with an interface temperature of 320 °C. For ionization, EI was employed at 70 eV.

## 5. Conclusions

We have developed a solvent-free screening method using Py/TD-GC–MS that enables simultaneous determination of restricted additives present at regulatory levels. The LODs of SCCPs, DecaBDE, HBCDD, and DEHP were less than 50 mg/kg, which is sufficiently lower than the regulatory criteria. With regard to reproducibility, an RSD of about 5% was confirmed by employing a spike recovery test on a polystyrene polymer solution containing the mixed standard solution. These results also represent a major step forward toward achieving simultaneous screening of harmful additives using Py/TD-GC–MS.

## Figures and Tables

**Table 1:** Target ions used for the quantification of simultaneous screening by Py/TD-GC–MS.

Additives	Target Ion (*m*/*z*)	Confirmation Ion (*m*/*z*)
SCCPs	89	115
HBCDD	319	561
DecaBDE	799	959
DEHP	279	149

**Table 2:** Summary of sensitivity, correlation coefficient (R^2^), and RSD.

Additives Name	Sensitivity (area/(mg/kg))	R^2^	RSD at 1000 mg/kg (%)	LOD (mg/kg)
DEHP	8.8 × 10^2^	0.998	5.0	14
SCCPs	5.9 × 10^2^	0.997	1.5	37
HBCDD	8.4 × 10	0.989	5.2	46
DecaBDE	2.6 × 10^2^	0.992	1.9	46

**Table 3:** Typical Py/TD heating conditions and GC–MS parameter settings.

Parameters	Settings
**Pyrolyzer**	
Furnace temperature	200 °C → 20 °C/min → 300 °C → 5 °C/min → 340 °C (1 min)
Interface temperature	300 °C
**GC**	
Column	100% Dimethyl polysiloxane, length 15 m, I.D. 0.25 mm, film, thickness 0.05 μm
Injection port temperature	320 °C
Column oven temperature	80 °C → (20 °C /min) → 300 °C (5 min)
Injection mode	Split (split ratio: 1/50)
Carrier gas	Helium, 52.1 cm/s, constant linear velocity
**MS**	
Ion source temperature	230 °C
Ionization method	EI, 70 eV

## Supplementary Materials

The following are available online. Table S1: Summary of experimental parameters; sensitivity, correlation coefficient (R^2^) and relative standard deviation (RSD) at 1000 mg/kg SCCPs solution, Table S2: Detection limit determined with reference to IEC62321-8, Table S3: LODs obtained by spiking mixed standard (ca. 200 mg/kg) to PS polymer matrix (0.5 mg).

## References

[1] Koch H.M., Preuss R., Angerer J.D. (2006). Di (2-ethylhexyl) phthalate (DEHP): Human metabolism and internal exposure—An update and latest results. Int. J. Androl..

[2] Ali T.E.-S., Legler J. (2010). Overview of the mammalian and environmental toxicity of chlorinated paraffins. Chlorinated Paraffins.

[3] Covaci A., Gerecke A.C., Law R.J., Voorspoels S., Kohler M., Heeb N.V., Leslie H., Allchin C.R., De Boer J. (2006). Hexabromocyclododecanes (HBCDs) in the environment and humans: A review. Environ. Sci. Technol..

[4] Shaw S.D., Berger M.L., Weijs L., Covaci A. (2012). Tissue-specific accumulation of polybrominated diphenyl ethers (PBDEs) including deca-BDE and hexabromocyclododecanes (HBCDs) in harbor seals from the Northwest Atlantic. Environ. Int..

[5] Alaee M., Arias P., Sjödin A., Bergman Å. (2003). An overview of commercially used brominated flame retardants, their applications, their use patterns in different countries/regions and possible modes of release. Environ. Int..

[6] The European Parliament and The Council of The European Union (2004). Regulation (EC) no 850/2004 of the European Parliament and of the Council of 29 April 2004 on Persistent Organic Pollutants and Amending Directive 79/117/eec. Off. J. Eur. Union.

[7] The European Parliament and The Council of The European Union (2007). Regulation (EC) no 1907/2006 of the European Parliament and of the Council of 18 December 2006, Concerning the Registration, Evaluation, Authorisation and Restriction of Chemicals (REACH). Off. J. Eur. Union.

[8] The European Parliament and The Council of The European Union (2011). Directive 2011/65/EU of the European Parliament and of the Council of 8 June 2011 on the Restriction of the use of Certain Hazardous Substances in Electrical and Electronic Equipment. Off. J. Eur. Union.

[9] The European Commission (2015). Commission Delegated Directive (EU) 2015/863 of 11 March 2015, Amending Annex II to Directive 2011/65/EU of the European Parliament and of the Council as Regards the List of Restricted Substances. Off. J. Eur. Union.

[10] Stringer R., Labunska I., Santillo D., Johnston P., Siddorn J., Stephenson A. (2000). Concentrations of phthalate esters and identification of other additives in PVC children’s toys. Environ. Sci. Pollut. Res..

[11] Lovekamp-Swan T., Davis B.J. (2003). Mechanisms of phthalate ester toxicity in the female reproductive system. Environ. Health Perspect..

[12] Darnerud P.O. (2003). Toxic effects of brominated flame retardants in man and in wildlife. Environ. Int..

[13] World Health Organization International Agency for Research on Cancer (1990). IARC Monographs on the Evaluation of Carcinogenic Risks to Humans.

[14] Eskilsson C.S., Björklund E. (2000). Analytical-scale microwave-assisted extraction. J. Chromatogr. A.

[15] Bart J.C. (2005). Additives in Polymers: Industrial Analysis and Applications.

[16] International Electrotechnical Commission (2017). IEC 62321-8, Determination of Certain Substances in Electrotechnical Products—Part 8: Phthalates in Polymers by Gas Chromatography-Mass Spectrometry (GC-MS), Gas Chromatography-Mass Spectrometry Using a Pyrolyzer/Thermal Desorption Accessory (Py/TD-GC-MS).

[17] Maruyama F., Fujimaki S., Sakamoto Y., Kudo Y., Miyagawa H. (2015). Screening of phthalates in polymer materials by pyrolysis GC/MS. Anal. Sci. Int. J. Jpn. Soc. Anal. Chem..

[18] Sobeih K.L., Baron M., Gonzalez-Rodriguez J. (2008). Recent trends and developments in pyrolysis-gas chromatography. J. Chromatogr. A.

[19] Yuzawa T., Watanabe C., Freeman R.R., Tsuge S. (2009). Rapid and simple determination of phthalates in plastic toys by a thermal desorption-GC/MS method. Anal. Sci. Int. J. Jpn. Soc. Anal. Chem..

[20] Rial-Otero R., Galesio M., Capelo J.-L., Simal-Gándara J. (2009). A review of synthetic polymer characterization by pyrolysis–GC–MS. Chromatographia.

[21] Tsuge S., Ohtani H., Watanabe C. (2011). Pyrolysis-GC/MS Data Book of Synthetic Polymers: Pyrograms, Thermograms and MS of Pyrolyzates.

[22] Fries E., Dekiff J.H., Willmeyer J., Nuelle M.-T., Ebert M., Remy D. (2013). Identification of polymer types and additives in marine microplastic particles using pyrolysis-GC/MS and scanning electron microscopy. Environ. Sci. Process. Impacts.

[23] Jansson K.D., Zawodny C.P., Wampler T.P. (2007). Determination of polymer additives using analytical pyrolysis. J. Anal. Appl. Pyrolysis.

[24] Kim J.W., Kim Y.M., Moon H.M., Hosaka A., Watanabe C., Teramae N., Choe E.K., Myung S.W. (2016). Comparative study of thermal desorption and solvent extraction-gas chromatography-mass spectrometric analysis for the quantification of phthalates in polymers. J. Chromatogr. A.

[25] Ballesteros-Gomez A., de Boer J., Leonards P.E. (2013). Novel analytical methods for flame retardants and plasticizers based on gas chromatography, comprehensive two-dimensional gas chromatography, and direct probe coupled to atmospheric pressure chemical ionization-high resolution time-of-flight-mass spectrometry. Anal. Chem..

[26] Kim Y.M., Kim J.W., Moon H.M., Lee M.J., Hosaka A., Watanabe A., Teramae N., Park Y.K., Myung S.W. (2017). Rapid quantification of N-methyl-2-pyrrolidone in polymer matrices by thermal desorption-GC/MS. Anal. Sci. Int. J. Jpn. Soc. Anal. Chem..

[27] Bart J. (2001). Polymer/additive analysis by flash pyrolysis techniques. J. Anal. Appl. Pyrolysis.

[28] The European Commission (2012). Commission Regulation (EU) no 519/2012 of 19 June 2012, Amending Regulation (EC) no 850/2004 of the European Parliament and of the Council on Persistent Organic Pollutants as regards Annex I. Off. J. Eur. Union.

[29] The European Commission (2015). Commission Regulation (EU) 2015/2030 of 13 November 2015, Amending Regulation (EC) no 850/2004 of the European Parliament and of the Council on Persistent Organic Pollutants as Regards Annex I. Off. J. Eur. Union.

[30] Fiedler H. (2010). Short-chain chlorinated paraffins: Production, use and international regulations. Chlorinated Paraffins.

[31] Tomy G.T. (2009). Analysis of chlorinated paraffins in environmental matrices: The ultimate challenge for the analytical chemist. Chlorinated Paraffins.

[32] Larsen E.R., Ecker E.L. (1988). Thermal stability of fire retardants:* III, decomposition of pentabromochlorocyclohexane and hexabromocyclododecane under processing conditions. J. Fire Sci..

[33] Takasuga T.M.H., Nouda C., Harada K., Koizumi A. Analysis of short-chain chlorinated paraffins (SCCPs) by GC-HRMS (NCI) with GC-HRToF-MS applied food sample. Proceedings of the 31st International Symposium on Halogenated Persistent Organic Pollutants (DIOXIN 2011).

[34] Castells P., Santos F.J., Galceran M.T. (2004). Evaluation of three ionisation modes for the analysis of chlorinated paraffins by gas chromatography/ion-trap mass spectrometry. Rapid Commun. Mass Spectrom..

[35] Junk S.A., Meisch H.U. (1994). Determination of chlorinated paraffins by GC-MS. Fresenius’ J. Anal. Chem..

[36] Zencak Z., Reth M., Oehme M. (2004). Determination of total polychlorinated *n*-alkane concentration in biota by electron ionization-MS/MS. Anal. Chem..

[37] Eric R.L., Ernest L.E. (1986). Thermal stability of fire retardants: I, hexabromocyclododecane (hbcd. J. Fire Sci..

[38] Pellizzato F., Ricci M., Held A., Emons H. (2007). Analysis of short-chain chlorinated paraffins: A discussion paper. J. Environ. Monit. JEM.

