# Supplementary material for: Simultaneous Screening of Major Flame Retardants and Plasticizers in Polymer Materials Using Pyrolyzer/Thermal Desorption Gas Chromatography Mass Spectrometry (Py/TD–GC–MS)
Source: Molecules. 2018 Mar 22;23(4):728. doi: 10.3390/molecules23040728 (PMC6017896; doi:10.3390/molecules23040728)
Supplement: Supplementary file 1 [file molecules-23-00728-s001.pdf]

molecules

Supplementary material

Simultaneous screening of major flame-retardants and plasticizers in polymer materials using pyrolyzer/thermal desorption gas chromatography mass spectrometry (Py/TD–GC-MS)

Hiroyuki Yanagisawa,[1] Yukihiko Kudo,[2] Katsuhiro Nakagawa,[2] Haruhiko Miyagawa,[2] Fumitaka Maruyama,[1] and Shigehiko Fujimaki[1], *

[1] *Consumer & Retail Service Division, SGS Japan Inc. YBP East Tower 12F, 134 Godo-cho, Hodogaya-ku, Yokohama 240-0005, Japan*

[2] *Analytical & Measuring Instrument Division, Shimadzu Corporation, 1 Nishinokyo Kuwabara-cho, Nakagyo-ku, Kyoto 640-8511, Japan*

* Correspondence: shigehiko.fujimaki@sgs.com; Tel.: +81-45-330-1101

Academic Editor: name
Received: date; Accepted: date; Published: date

Supplementary material Table S1**.** Summary of experimental parameters; sensitivity, correlation coefficient (R^2) and relative standard deviation (RSD) at 1,000 mg/kg SCCPs solution.

Fragment ion (*m*/*z*)	Sensitivity (Area/(mg/kg))	R^2	RSD (%)
75	7.7×10^2	0.998	5.2
89	8.8×10^2	0.998	5.0
91	6.1×10^2	0.999	4.8
105	4.2×10^2	0.999	3.0
115	3.0×10^2	0.999	4.0
125	2.8×10^2	0.998	5.0
151	2.4×10^2	0.999	6.0

Supplementary material Table S2. In accordance with the provisions of IEC62321-8 (student's t-statistic with 99% confidence,), LODs were determined by multiplying t (n-1, risk rate) =3.14 (risk rate = 0.01) to each the standard deviation.

Number of test	DEHP	SCCPs	HBCDD	Deca-BDE
n=1	126	103	136	104
n=2	121	100	138	84
n=3	118	111	129	128
n=4	119	118	121	98
n=5	120	113	115	103
n=6	117	125	122	115
n=7	112	130	98	103
Average	119	114	123.	105
Standard deviation (t)	4.23	11.1	13.8	13.7
LOD/ (mg/kg)	14.2	37.3	46.4	46.0

Supplementary material Table S3. LODs obtained by spiking mixed standard (ca. 200 mg/kg) to PS polymer matrix (0.5 mg). In accordance with the provisions of student's t-statistic with 99% confidence, LODs were determined by multiplying t(n-1, α) =4.54 (α= 0.01) to each the standard deviation of 4 repeated measurements.

	DEHP	SCCPs	HBCDD	DecaBDE
LOD/ (mg/kg)	53	56	52	42
